# The relationship of cytomegalovirus with physical functioning and health-related quality of life in older adults

**DOI:** 10.1007/s41999-025-01244-6

**Published:** 2025-06-13

**Authors:** Frances A. Kirkham, Phu Sabei Shwe, Ekow Mensah, Chakravarthi Rajkumar

**Affiliations:** 1 Department of Clinical and Experimental Medicine, Brighton and Sussex Medical School, University of Brighton and University of Sussex, Brighton, BN1 9PX UK; 2 Department of Stroke Medicine, University Hospitals Sussex NHS Foundation Trust, Royal Sussex County Hospital, Brighton, BN2 5BE UK; 3 Monash Ageing and Research Centre (MONARC), Melbourne, 3168 Australia

**Keywords:** Cytomegalovirus, Sarcopenia, Frailty, Quality of life

## Abstract

**Aims/introduction:**

Cytomegalovirus (CMV) is a highly prevalent virus, known to be associated with cardiovascular morbidity. It has been hypothesised to play a role in the development of frailty, but its association with physical function and sarcopenia has yet to be fully understood. This study aimed to look at the relationship between CMV, sarcopenia and health-related quality of life in older adults.

**Methods:**

210 people in the south of England, UK, with median age 68 years (49% male, 51% female) underwent demographic and medical questionnaires, measures of body composition (bioimpedance analysis), handgrip strength and the short form 36 item survey of quality of life (SF36). All participants had CMV serology and inflammatory markers measured. Measures of sarcopenia were calculated using European Working Group definitions.

**Results:**

51.7% of participants were positive for CMV Immunoglobulin G (IgG). There were no significant differences between CMV positive and negative groups in age, gender, measures of sarcopenia or inflammatory markers. CMV positive groups had lower scores in all domains of the SF36, with significantly lower physical function score (88.7 vs 81.3, *p* = 0.003) as well as limitations due to physical health, energy/fatigue, social functioning and pain. On linear regression, CMV status was significantly associated with SF36 physical function score (*p* = 0.004) after adjustment for age, gender, body mass index, C-reactive protein and handgrip strength.

**Conclusions:**

CMV positivity is significantly associated with physical function and health-related quality of life in older adults, although its direct relationship to sarcopenia is yet to be fully ascertained.

**Supplementary Information:**

The online version contains supplementary material available at 10.1007/s41999-025-01244-6.

## Key summary points

**Aims:**

The aim of this paper is to show the relationship between cytomegalovirus with physical functioning and health related quality of life in older adults.

**Findings:**

CMV status is independently associated with physical functioning, as measured by the SF36.

CMV positive groups had lower values on all domains of health-related quality of life compared to CMV negative groups.

SF36 physical functioning correlates with measures of sarcopenia and may represent a way of detecting early signs of sarcopenia.

**Message:**

This paper adds to our understanding of the role CMV exposure plays in changes to muscle mass and function and the subsequent development of sarcopenia.

This paper shows that interventions to reduce the burden of CMV (e.g. vaccines) may have a role in reducing subsequent sarcopenia.

This paper shows that SF36 measures of physical function may be a method by which precursors of sarcopenia may be detected in this cohort.

**Supplementary Information:**

The online version contains supplementary material available at 10.1007/s41999-025-01244-6.

## Introduction

Cytomegalovirus (CMV) is a pro-inflammatory herpes virus that is highly prevalent across the world and can establish chronic infections. CMV has been associated with cardiovascular disease [1], cognitive decline and mortality [2]. It is hypothesised to contribute to chronic inflammation and immunosenescence. To date, most studies have focused on the impact of CMV on vascular and endothelial smooth muscle, looking to explain its harmful impact on the cardiovascular system [3].

Sarcopenia is defined as loss of muscle mass with age beyond that due to reduced physical activity. It has been an increasing area of research due to its association with the frailty syndrome, poor health outcomes and mortality [4–6]. Evidence is growing to suggest that clinical interventions may reduce the negative consequences of sarcopenia, but these are not routinely offered. There remain difficulties defining thresholds for sarcopenia and creating a globally accepted consensus on diagnosis and management, although progress has been made recently [7]. The most recent European Working Group definition (EWGSOP2) focuses on muscle function rather than mass, partly due to difficulty and lack of routine measurement of muscle mass [8]. Grip strength is inexpensive to perform, non-invasive and can be easily incorporated into routine practice [9]. Measures of muscle mass are used to confirm diagnosis, and bioimpedance analysis is accepted as equivalent in accuracy to DEXA or CT measurements [10].

There have been numerous studies linking CMV to general frailty and a small number investigating its relationship to sarcopenia [31,32]. Few studies to date have investigated the relationship between grip-strength and CMV status; one finding no association, but suggesting there may be an association between muscle cross-sectional area and CMV status in men only, [11], while another found an association between higher CMV IgG levels and reduced grip strength in men [12]. In people living with HIV, CMV positivity has been shown to be linked to worse physical performance but not muscle density [13]. Physical function has been shown to be a strong correlate of measures of quality of life [14], but the implications of sarcopenia and reduced muscle mass on quality of life and physical functioning have yet to be investigated in the context of CMV positivity.

This study aimed to explore the relationship between CMV status, sarcopenia and health-related quality of life in a cohort of over 60s in the United Kingdom. We sought to investigate the association of CMV exposure with reduced muscle mass and reduction in physical function and quality of life.

## Methods

People aged 60 years or over were recruited through the primary care research network and general practices in Southeast England. Inclusion criteria: White British and age 60 years or older. Exclusion criteria: pre-existing immunodeficiency, organ transplant, use of immunosuppressive or immune-modulating drugs in the last year, cancer or treatment for cancer in the last 5 years, insulin-dependent diabetes, moderate or advanced renal failure, liver disease, endocrine disorders (except corrected thyroid dysfunction), manifest autoimmune disease, dementia/lack of capacity to consent, known alcoholism or other drug abuse, acute infection or illness in the last 4 weeks, raised body temperature (> 37.5 °C), moderate or severe heart failure (NYHA III or IV), inability to lie flat.

Participants were asked for a detailed medical history, including demographic and lifestyle factors and medications. They underwent physical examination, height, weight, blood pressure measurement and assessment of vascular stiffness by the cardio-ankle vascular index (CAVI—VaSera VS-1500 N, Fukuda Denshi, Tokyo, Japan). Blood samples were taken by peripheral venepuncture and CMV immunoglobulin G (IgG) serology (Architect CMV IgG, Abbot, Maidenhead, UK) was performed at the Brighton and Sussex University Hospital Trust virology laboratory using a routine assay. Participants whose serum samples exceeded the assay threshold for positive IgG are referred to as 'CMV positive’. C-reactive protein (mg/l) was also measured.

Grip strength was measured using a Takei hand dynamometer (Takei Kiki Kogyo, Tokyo, Japan) as the average of three readings from the dominant hand. Grip strength was also adjusted for BMI to give an alternative measure. Cut-offs for reduced grip strength were defined in British cohorts by Dodds et al [15]. Studenski’s cut-offs for adjusted grip strength were also used [16]. Cut-offs are presented in Supplementary Material Table 1.

**Table 1:** Comparison of baseline characteristics between total cohort and CMV positive and negative groups (p values based on independent samples *t* test)

	CMV negative	CMV positive	*p* value
Age in years	69.5 (7.5)	70.0 (7.1)	0.614
Gender (%f:m)	51:49	51:49	0.936
Combined pack years	8.3 (13.4)	9.8 (17.5)	0.499
Systolic Blood Pressure (lying) (mmHg)	138.9 (17.5)	136.1 (18.1)	0.261
Diastolic Blood Pressure (lying) (mmHg)	76.9 (9.5)	75.5 (10.6)	0.336
Average Cardio-Ankle Vascular Index	9.23 (1.1)	9.28 (1.3)	0.775
C-Reactive Protein (mg/l)	2.63 (6.46)	2.78 (5.75)	0.864
Handgrip strength (kg)	26.9 (11.0)	27.7 (10.4)	0.598
Appendicular skeletal muscle mass (kg)	21.0 (5.5)	21.0 (4.8)	0.972
Appendicular skeletal muscle mass index	0.79 (0.17)	0.80 (0.18)	0.818

Bioimpedance analysis (BC-418, Tanita, Tokyo) was used to measure body composition. Appendicular skeletal muscle mass (ASM) was assessed and appendicular skeletal muscle mass index (ASMI) was calculated, adjusting ASM for height squared. An alternative measure of ASMI was calculated by adjusting ASM for BMI, as recommended by Studenski et al [16]. Reduced ASM was defined using the Sergi Eq. [17,18] and reduced ASMI was defined using the FNIH sarcopenia project, based on a large European population [16] (Supplementary Material Table 1).

European Working Group (EWGSOP2) definitions of sarcopenia were used with probable sarcopenia defined by reduced grip strength and definite sarcopenia defined by the addition of reduced ASM or ASMI. The EWGSOP2 also included a further characterization of severe sarcopenia with the addition of reduced physical performance as measured by a timed up and go test or gait speed, however these measures were not taken in our cohort.

To assess health-related quality of life and self-reported physical functioning, the RAND 36 item short-form survey (SF36 version 1.0) was performed on all participants and scored according to the creators' guidelines [19] to give overall scores and scores in seven domains: physical functioning, role limitations due to physical health, role limitations due to emotional health, energy/fatigue, emotional wellbeing, social functioning, pain and general health. Answers given are scored from 0 to 100 and relevant questions combined. Higher scores indicate better quality of life. The SF36 has been shown to be equal in performance to other widely used measures of quality of life [20].

### Ethics statement

The study was approved by the UK National Research Ethics Service (NRES) 'London Centre' (Reference 13/LO/1270). Written informed consent was obtained from all participants. The study was conducted in accordance with the Declaration of Helsinki.

### Statistical analysis

Statistical analysis was performed using SPSS v29.0.1.0 (IBM, Armonk, NY), with *p* < 0.05 considered statistically significant. Values are expressed as mean ± standard deviation. Independent samples t-test was used to compare baseline characteristics between participants according to CMV status and sarcopenia status. Linear regression was performed to determine whether SF36 physical functioning score (dependent variable) was associated with CMV status independent of potential confounders (smoking history, age, gender, BMI). Bivariate correlation was used to evaluate the correlation between variables.

## Results

210 people took part with mean age 69.8 years (± 7.3), including 108 women (51%) and 102 men (49%). Past medical history: a small proportion of the cohort reported previous medical issues: previous stroke — 2 (1%), previous Transient Ischaemic Attack — 5 (2%), hypertension – 74 (36%), atrial fibrillation – 18 (9%), congestive heart failure — 4 (2%), ischaemic heart disease — 11 (5%), Chronic Obstructive Pulmonary Disease — 3 (1%), malignancy — 13 (6%), arthritis — 47 (23%), diabetes mellitus — 11 (5%), hyperlipidaemia — 58 (28%). In terms of lifestyle factors, 7% of the cohort were current smokers and 49% were ex-smokers.

108 participants had positive CMV serology (51.7%). Table 1 compares characteristics between the total cohort and CMV positive and negative groups. There were no statistically significant differences between CMV groups in terms of age, gender, smoking history, blood pressure, arterial stiffness (measured by CAVI) or CRP. There were no significant differences in measures of sarcopenia between CMV groups.

Prevalence of sarcopenia varied depending on the definition used. According to the EWGSOP2 definition of probable sarcopenia, prevalence was 23.9% overall, with 24.5% for females and 23.2% males. Definite sarcopenia had a prevalence of 8.8% overall, with 7.5% of females and 10.2% of males meeting this definition. When using measures of either muscle strength or muscle quantity, there was a prevalence of sarcopenia of 31.2% overall, with 31.1% for females and 31.3% for males. There were no statistically significant differences in handgrip strength, adjusted handgrip strength, ASM or ASMI between CMV positive and negative groups for either males or females (supplementary material Table 2).

**Table 2:** Comparison in SF36 scores between CMV negative and positive groups on independent samples *t* test

	CMV negative	CMV positive	P value
Physical functioning	**88.7 (15.0)**	**81.3 (20.8)**	0.003**
Limitations due to physical health	**89.1 (29.0)**	**77.8 (36.7)**	0.015*
Limitations due to emotional health	95.0 (18.5)	90.4 (25.0)	0.132
Energy/fatigue	**74.9 (16.1)**	**69.2 (19.1)**	0.022*
Emotional wellbeing	87.4 (9.9)	84.1 (14.3)	0.056
Social functioning	**93.9 (13.1)**	**88.3 (18.9)**	0.014*
Pain	**87.3 (16.7)**	**78.1 (24.0)**	0.002**
General health	75.6 (15.2)	73.7 (18.8)	0.403

*Indicates *p* < 0.05

**Indicates *p* < 0.01

There were significant differences in SF36 scores between CMV groups in the following domains: physical functioning, limitations due to physical health, energy/fatigue, social functioning and pain (Table 2). CMV positive groups had lower values in all SF36 domains compared to CMV negative groups.

Bivariate correlation was performed to assess correlation of SF36 domains with other variables. Physical functioning was significantly correlated with age, BMI, handgrip strength and ASMI. Other significant correlations are highlighted in Table 3.

**Table 3:** Bivariate correlations of SF36 domains with age, BMI and measures of sarcopenia

	Age	BMI	Handgrip strength	ASM	ASMI
Physical functioning	*r* = − 0.284** ***p* = < 0.001**	*r* = − 0.152* ***p* = 0.028**	*r* = 0.155* ***p* = 0.026**	*r* = 0.099 *p* = 0.164	*r* = 0.201 *p* = 0.005**
Limitations due to physical health	*r* = − 0.122 *p* = 0.079	*r* = − 0.024 *p* = 0.733	*r* = 0.070 *p* = 0.320	*r* = 0.089 *p* = 0.210	*r* = 0.099 *p* = 0.163
Limitations due to emotional health	*r* = − 0.043 *p* = 0.535	*r* = − 0.104 *p* = 0.136	*r* = 0.024 *p* = 0.734	*r* = 0.069 *p* = 0.337	*r* = 0.131 *p* = 0.066
Energy/fatigue	*r* = − 0.099 *p* = 0.155	***r* = − 0.147** *p* = 0.033*	*r* = 0.056 *p* = 0.426	*r* = 0.048 *p* = 0.503	*r* = 0.010 *p* = 0.122
Emotional wellbeing	*r* = 0.008 *p* = 0.910	*r* = − 0.041 *p* = 0.552	*r* = 0.124 *p* = 0.077	*r* = 0.135 *p* = 0.059	*r* = 0.156 *p* = 0.028*
Social functioning	*r* = − 0.202** ***p* = 0.003**	*r* = − 0.150* ***p* = 0.030**	*r* = 0.083 *p* = 0.236	*r* = 0.088 *p* = 0.219	*r* = 0.169 *p* = 0.017*
Pain	*r* = − 0.032 *p* = 0.643	*r* = − 0.096 *p* = 0.169	*r* = 0.033 *p* = 0.634	r = 0.085 *p* = 232	*r* = 0.140 *p* = 0.050
General health	*r* = − 0.070 *p* = 0.310	*r* = 0.003 *p* = 0.964	*r* = − 0.062 *p* = 0.376	*r* = − 0.028 *p* = 0.700	*r* = − 0.046 *p* = 0.515

*Indicates *p* < 0.05

**Indicates *p* < 0.01

Linear regression was performed to assess whether CMV status contributes to physical functioning score of SF36 independent of other variables (smoking, age, sex, BMI, CRP and handgrip strength). CMV status remained a significant contributor to physical functioning score (*p* = 0.003) with smoking history, age and BMI also remaining significant (Table 4).

**Table 4:** Linear regression with physical functioning domain of SF-36 as dependent variable

Independent variables	Unstandardised B	Significance
Smoking pack years	− **0.313**	< 0.001**
Age	− **0.694**	< 0.001**
Gender	4.260	0.228
BMI	− **0.761**	0.011*
CRP	− 0.094	0.630
CMV status	− **6.938**	0.004**
Handgrip strength	0.064	0.706

R squared for model = 0.232, *F* = 8.216, *p* < 0.001

*Indicates *p* < 0.05

**Indicates *p* < 0.01

## Discussion

Our results show a clear relationship between CMV status and health-related quality of life, particularly the physical domains, as measured in the SF36 survey. However, while the physical functioning domains of the SF36 correlate with measures of sarcopenia, there was no direct association of CMV status with measures of sarcopenia.

Our study found an association between CMV positivity and reduced health-related quality of life in the following domains: physical function, limitations due to physical health, energy/fatigue, social functioning and pain. A Finnish study of 383 patients with cardiovascular disease found that higher CMV antibody titre was associated with increased comorbidity, functional impairment and reduced health-related quality of life, as measured by the 15D instrument [21]. The results do not separate the different elements of quality of life, which is an advantage of the SF36 in showing how CMV may impact health-related quality of life. A case control study of people living with HIV found that CMV antibody titres were associated with functional impairment (measured through the Short Physical Performance Battery and Fried’s frailty index), which was not true for other herpesviruses. The authors suggest that inflammation and T cell activation may be the mechanism by which CMV acts to impair functional ability in this population. Similarly, in a population of older nursing home residents, an association of CMV antibody levels with reduced functional ability (measured by the Barthel’s Index) was found, postulating that immune cell activation and aging of the immune system undergo complex interactions resulting in clinically apparent impairment in function [22]. In a large study of older Latinos, impaired functional ability was associated with both CMV and CRP levels, reinforcing the notion that inflammation is a key mediator [23]. However, the association disappeared after adjusting for number of health conditions, BMI and household income which may add to the theory that CMV increases certain co-morbidities and this is how it reduces physical function.

We did not find any association between the mental health components of quality of life and CMV. There is mixed evidence on this subject, with some studies suggesting a link between CMV and incident depression [24]. While others failed to find an association after adjustment for confounders [25]. It is likely that CMV has a widespread impact on the body, associated with multiple conditions which can impair daily function and quality of life, thus impacting mental health. In terms of clinical applicability, our data suggest that CMV has an impact on health leading to reduced quality of life. Although it is unclear which specific conditions may be impacted and, therefore, improved by intervention, from a patient perspective, quality of life is one of the most important factors and thus understanding the role of CMV has the potential to aid us in designing interventions that may prevent this impairment. Further studies are required to assess the exact impact of CMV over time and how it contributes to physical function reduction.

The majority of previous studies have focused on quality of life in the context of general frailty, often using SF36 [26,27]. In the context of CMV, there have been limited studies looking at the relationship of CMV status and frailty, with different measures of frailty used. CMV reactivation was shown to be associated with an odds ratio of 6.13 for frailty compared to non-frail Brazilian women [28].Schmaltz et al. found higher levels of frailty in CMV positive older women, suggesting this association may be modified by IL6, as a higher association was found in those with higher IL6 levels [29]. However, they were not able to show a statistically significant association of CMV status with grip strength, although there was a trend towards increased weakness. Another study of women found an association between CMV status and both frailty (hazard ratio 3.45, 95% confidence interval 1.45–8.27) and mortality (HR 2.79, 95% CI 1.64–8.83), but only the highest quintile of antibody titres remained significant in predicting mortality after adjustment and the association with frailty became non-significant [30]. Similarly, using Fried’s criteria, a Belgian study of the oldest old found no association between pre-frailty and CMV status with only IL6, age and sex found to be predictors of the pre-frail phenotype [31].The BELFRAIL study, also of over 80s in Belgium, counterintuitively found a negative association of CMV serology with frailty [32]. They suggest that a survival effect may result from the older age of their population, meaning that those more susceptible to the damage of CMV have died at earlier ages. They also found no association of CMV status with IL6 levels, although IL6 itself was associated with frailty and functional impairment. The level of association of CMV with all elements of frailty is significant, as there is increasing belief that frailty is to some degree reversible at the pre-frail stage, thus clinical interventions may have a role to play in reducing the burden of frailty.

The SF36 is a commonly used measure of health-related quality of life but is not specific to older people and has some limitations. Currently, there are no defined thresholds to identify reduced physical function, although cut offs for some mental health components have been proposed [33]. To give our figures some context, one large study used the US general population as a baseline for comparison, finding that the mean physical function score was 90 (± 17) [34]. In a much younger population of healthy controls (mean age 30–40), the mean physical functioning score was 92.6 ± 14.2 [35]. It would appear reasonable, given the older average age of our cohort, that the CMV negative group had a slightly lower score (88.7 ± 15) and shows that the CMV positive group had a significantly lower value (81.3 ± 20.8). It has been determined that the minimal clinically impactful difference in the SF36 is a difference of 3–5 on each scale [19], thus the differences highlighted in this study represent clinically impactful figures.

SarQoL is a sarcopenia-specific measure of quality of life, which has been shown to correlate well in detecting robust vs frail vs prefrail individuals [37]. However, one study found that the SF36 was similarly able to detect differences between patients and controls when compared to SarQoL and was found to be superior to SARC-F [36], a survey designed for use in case-finding for sarcopenia. This study is focused on identifying individuals who may be vulnerable to the detrimental effects of CMV exposure and so finding diagnostic tools that highlight such individuals at an early stage (before clinical symptoms have developed) could be of benefit to clinicians. The SF36 provides a simple, effective tool for this purpose and further studies to validate its use in this context are needed.

The prevalence of sarcopenia in our cohort mirrors that found in previous European studies. Our prevalence of 8.8%, according to the full EWGSOP2 definition of reduced muscle function and mass, is similar to that found in the large FNIH study of ambulatory males (3–8%) [38]. In a significantly older cohort, reduced grip strength was found in 23% of males and 26.6% of females [15], while our younger cohort demonstrated reduced grip strength in 23.2% of males and 24.5% of females. Loss of strength has been shown to be an accurate predictor of future worsening mobility [39].

This study did not find a significant difference in handgrip strength or measures of sarcopenia between CMV groups. However, this study did show significant differences in SF36 scores between CMV groups, particularly in physical function. This may suggest that CMV exposure has an impact on physical function through a mechanism other than measurable changes in muscle strength or mass. Previous research has demonstrated a relationship between health-related quality of life and sarcopenia. Low grip strength (in both sexes) was found to be associated with poor scores on all SF36 domains, remaining associated with reduced physical functioning after adjustment [40]. One Spanish study of predominantly older women found that probable sarcopenia was associated with lower SF36 domains, including physical functioning [41]. Interestingly, the associations were less pronounced for participants with definite sarcopenia, and thus the impairment of physical functioning associated with CMV exposure may act through other mechanisms than via sarcopenia. However, the aforementioned study used cut offs for sarcopenia based on Skeletal Mass Index (SMI) taken from community-dwelling older people in Taiwan. These cut offs may be inappropriate in a European population which may not have comparable characteristics in terms of body composition. Inevitably, sarcopenic individuals are likely to have reduced physical functioning and thus lower scores on this element of the SF36. A small longitudinal study found that declining physical quality of life, defined by SF36 domains, was associated independently with physical performance and muscle cross-sectional area over 3-year follow up [42]. This suggests that the SF36 may be a useful early indicator of worsening mobility and subsequent muscle loss, underlining the importance of interventions to maintain skeletal muscle health in older populations. It is unclear from our results whether CMV exposure leads to changes in physical function that may predate measurable changes in muscle mass and further longitudinal studies are required to consider this possibility,

Inflammation and immunosenescence have been proposed as mechanisms by which CMV may contribute to reduced physical function. One study identified a ‘cluster’ of the oldest old with an immunosenescent profile which included CMV positivity and T cell senescence [43]. However, similarly to ours, this study failed to find a relationship to muscle loss or sarcopenia over 5 years. It is debatable whether 5 years is sufficient to detect changes relating to CMV exposure, which is likely to have occurred in childhood or adolescence. There was also a 40% loss to follow up which may have underpowered the final results. In a similar population of the oldest old, there was limited evidence to support immunosenescence as the key element driving the association of frailty and inflammation, with no association found between CMV status and frailty. This may support the idea of a survival effect in this older group [44]. Equally, the duration of CMV infection does not seem to affect the impact on the system [45], suggesting that the damage may relate to some vulnerability factor in the host.

Changes to the immune system occurring over time include reduced capacity to fight infection and increased inflammation (‘inflammageing’), which are postulated to relate to higher levels of IL6. In one study, IL6 levels strongly predicted grip-strength, suggesting a possible role in reducing physical function. However, there is the possibility raised by authors that cardiovascular disease may be a mediating factor, resulting in less physical activity. It is also unclear whether CMV reactivation is the cause or effect of immunological changes with age. There is inevitably a cumulative impact of changes in nutrition, activity levels, endocrinological change and inflammatory changes contributing to impaired function, frailty and its component parts [46].

Some studies have suggested that CMV may contribute to immunosenescence through clonal proliferation of terminally differentiated CMV-specific T cells, alongside chronic inflammation leading to persistent accumulation of inflammatory damage [47,48]. We have previously written about the increase in T memory cells, predominantly in men, in the CMV positive group of this cohort [49]. There is also the possibility of a dose–response relationship, with higher CMV IgG more strongly correlated with frailty [50]. Chronic low-grade inflammation may be a response to chronic or latent infections such as CMV, resulting in higher levels of circulating inflammatory markers including CRP. This has mainly been studied in people living with HIV where CMV infection is highly prevalent and CMV is the target of over 10% of circulating T cells in people with HIV [51].

This was a cross-sectional study of British older people and thus cannot infer causality and may not be generalisable to other populations. Recruitment was limited to White British people to assess the impact of CMV in a specific cohort of the population; however, this means that results are not fully representative of the overall British population. There are proven ethnic differences between Caucasian and African-Caribbean and Asian groups in vascular risk factors. The main study looked at relationship between CMV status and cardiovascular risk hence we included only one ethnic group to avoid this as a confounding factor. Interestingly, one US cohort study of CMV found that seroprevalenvce was associated with increased hazard ratio for all-cause mortality in non-hispanic white people but not other ethnic groups [52]. Our exclusion criteria included a number of significant comorbidities to avoid confounding the influence of CMV; however, it is possible these exclusions reduce the generalisability of results. The use of a linear scale to detect differences in SF36-assessed physical function offers advantages to simply categorising participants into frail/non-frail or sarcopenic/non-sarcopenic, although it has limitations in only assessing generic health-related quality of life and is not condition-specific. We chose to use recommended measures and cut-offs for muscle quality and quantity based on European guidelines. BIA is recommended for use in assessing body composition but does have limitations as it is dependent on hydration and relies on specific population norms for comparison. The use of CMV antibodies also may not be sufficient to characterise the status of a large population, as 1 in 4 people may fail to generate a humoral response. In this case, it would be necessary to measure cellular immunity^25^.

## Conclusion and future directions

This study found an independent association of physical function domains of the SF36 with CMV status, but no clear relationship between CMV status and sarcopenia. Previous studies have suggested that CMV may contribute to overall frailty, but the relationship with muscle loss over time is yet to be determined. Future studies would be beneficial to assess the relationship of changes in measures of physical function with other effects of CMV in this cohort, thus offering the potential for interventions to reduce the negative consequences. The exact mechanism by which frailty, immune senescence and CMV-related inflammation are linked remains uncertain, likely due to the complex, heterogeneous interactions between these states. It is possible that better understanding of the role of CMV in the impairment of physical elements of quality of life may have a role in reducing the future burden of frailty, but further elucidation of these relationships is required.

## Supplementary Information

Below is the link to the electronic supplementary material.

Supplementary file1 (DOCX 13 KB)

## Data Availability

The datasets generated and analysed during the study are available on request to the corresponding author.
