# Supplementary material for: The relationship of cytomegalovirus with physical functioning and health-related quality of life in older adults
Source: Eur Geriatr Med. 2025 Jun 13;16(5):1851–9. doi: 10.1007/s41999-025-01244-6 (PMC12528222; doi:10.1007/s41999-025-01244-6)
Supplement: Supplementary file 1 — Supplementary file1 (DOCX 13 KB) [file 41999_2025_1244_MOESM1_ESM.docx]

Supplementary material Table 1 Table presenting cut offs used in defining sarcopenia measures for each sex

| Variable | Cut off for males | Cut off for females |
| --- | --- | --- |
| Reduced grip strength | <27 kg | <16 kg |
| Reduced adjusted grip strength | <0.1 | <0.56 |
| Reduced appendicular skeletal muscle mass | <20kg | <15kg |
| Reduced appendicular skeletal muscle mass index | <0.789 | <0.512 |

Supplementary material Table 2 Table comparing measures of sarcopenia between CMV positive and negative groups for each sex

|  | Females | | | Males | |
| --- | --- | --- | --- | --- | --- |
| Variable | CMV - | CMV + | P value | CMV - | CMV + |
| Handgrip strength (kg) | 19.3 (5.1) | 20.4 (6.8) | NS | 35.4 (9.5) | 35.3 (7.7) |
| Handgrip strength/BMI | 0.76 (0.22) | 0.80 (0.30) | NS | 1.3 (0.4) | 1.3 (0.4) |
| Appendicular skeletal muscle mass (ASM) (kg) | 16.8 (3.0) | 17.3 (2.2) | NS | 25.5 (3.6) | 24.9 (3.4) |
| Appendicular skeletal muscle mass index (ASMI) | 0.67 (0.11) | 0.66 (0.08) | NS | 0.93 (0.10) | 0.94 (0.14) |
